# “I want to talk about it, but at the same time, I don't want to, so it's just easier to draw it”: perceived benefits of an arts-based intervention to support children's basic psychological needs and well-being

**DOI:** 10.3389/frhs.2026.1920816

**Published:** 2026-08-28

**Authors:** Jasmine Piché, Fahim Moominzada, Zachary Dylan Fry, Sandrine Corbeil, Laurie Deschenes, Emilie Mclean, Kyra Simons, Adrianna Mendrek, Catherine Malboeuf-Hurtubise

**Affiliations:** 1 Department of psychology, Université de Sherbrooke, Sherbrooke, Qc, Canada; 2 Department of psychology, Bishop's University, Sherbrooke, Qc, Canada; 3 School of psychology, University of Wollongong, Wollongong, NSW, Australia; 4 School of psychology, Université Laval, Québec City, Qc, Canada; 5 Department of psychology, Université du Québec à Montréal, Montreal, Qc, Canada; 6 Department of psychology, Wilfrid Laurier University, Waterloo, On, Canada

**Keywords:** arts-based intervention, children mental health, psychological need satisfaction, self-determination, well-being

## Abstract

Children from low-income families and rural areas are particularly vulnerable to developing emotional and behavioral problems. Implementing mental health intervention into after-school programs can serve as an opportunity to foster the well-being of these children. Arts-based interventions appear particularly effective at engaging children in reflections on daily challenges and processing associated emotions and thoughts, thus supporting their well-being.

**Study Aims:**

In collaboration with an after-school program, this study aimed to explore the perceived benefits of an arts-based intervention on children’s well-being and document how the intervention supported their self-determination.

**Methods:**

An 8-week arts-based intervention was implemented in an after-school program. Using a qualitative design, semi-structured interviews (*n*=14) were conducted with the participants and the after-school program coordinators (*n*=1) to capture their perspectives of the intervention. Focus groups and observational data were used to enrich the data from the interviews. Inductive-deductive thematic analyses were used to document the emerging themes.

**Results:**

Two main themes emerged from the interviews with the coordinators. First, the intervention fostered the development of positive and secure relationships, between the research team and the particpants, among participants themselves, and between participants and program coordinators. Second, the intervention provided an opportunity to develop socioemotional competencies and coping skills. Three main themes emerged from the interviews with the participants. The intervention provided a safe and supportive environment where children were able to develop self-awareness and consciousness of others, as well as foster emotional expression and positive affect. Moreover, the intervention supported, to some extent, children’s basic psychological needs for autonomy, competence, and relatedness

**Implications:**

These findings provide support to the existing literature on arts-based interventions in fostering children mental health. This type of intervention appeared to be particularly beneficial for underprivileged children, as it is non-threatening and less confronting than other mental health interventions.

## Introduction

Children today are exposed to a wide range of stressors, from academic pressures to social challenges, that can significantly affect their well-being and developmental trajectories (1). Indeed, over the past decades, mental health problems among youth have increased substantially (2). Findings from the *2022 health behaviour school-aged children study* revealed that in Canada, the proportion of children in grades 6 to 10 who reported feeling sad, hopeless, lonely, and anxious has risen considerably since the early 2000s, while self-confidence and life satisfaction have declined (3).

These challenges may be particularly pronounced among children from disadvantaged backgrounds, who may be more frequently exposed to physical and psychosocial stressors (i.e., familial adversity, financial hardship, and food insecurity) (4). Exposure to adversity in early life has been associated with a range of negative psychological and developmental outcomes, and evidence suggests that youth growing up in disadvantaged contexts may be at heightened risk of developing mental health problems (5, 6). Socioeconomic disadvantage has also been associated with educational and developmental disparities, with children from low-income families more likely to experience academic difficulties and early school dropout than their more privileged peers (5). Given these inequities, there is a need to support these youth to prevent long-term mental health consequences from persisting into adulthood.

Despite experiencing greater exposure to mental health risk factors, children from disadvantaged backgrounds may have limited access to mental health services (7). Multiple barriers can affect the availability, accessibility, and uptake of appropriate support for these children, including shortages of mental health professionals, financial constraints, cultural barriers, geographic isolation, and stigma. These obstacles contribute to disparities in access to care and may further exacerbate mental health challenges, particularly in rural communities (8). In rural communities, negative cultural attitudes and beliefs surrounding mental health may discourage people from seeking help, further limiting access to needed support (8).

One promising approach to reducing these barriers and improving accessibility is through universal preventative and health-promoting measures, offered in school or after-school settings (9). These initiatives focus on developing socioemotional competencies, emotional regulation strategies, emotional literacy, and resilience as protective factors, reaching larger populations and therefore reducing gaps in mental health services.

### After-school programs

After-school programs represent an important resource for supporting children's cognitive, emotional and social development. Typically offered by community organizations, youth associations, or schools as an extension of regular school activities, these programs bridge the school and home environment and can contribute to educational success and positive youth development (10). After-school programs provide opportunities for academic enrichment and recreational activities, including sports and arts, which may help address educational disparities and promote positive development, particularly among children from disadvantaged families (4, 11). Beyond academic support, after-school programs create opportunities for socialization, foster positive relationships with peers and staff, and promote the development of social and emotional competencies (12). Evidence suggests that strengthening social support and socioemotional competencies may have a protective effect on disadvantaged youth (9). As such, after-school programs may be uniquely positioned to promote children's well-being, making them a strategic setting for delivering mental health interventions (4, 12). Initiatives such as positive youth development (PYD) and social-emotional learning (SEL) programs have demonstrated potential to support children's positive development and well-being (13).

Among the activities offered in after-school programs, art activities may be particularly well suited to support children's psychological development. Arts can provide opportunities for children to reflect on everyday challenges and express or process associated emotions and thoughts in developmentally accessible ways (14). Findings from a quasi-experimental study demonstrated positive mental health outcomes among vulnerable youth participating in an arts after-school program (15). Indeed, art may be an accessible medium for mental health promotion, as it can be perceived as less intimidating than traditional talk-based therapies. This may help reduce stigma and resistance associated with seeking mental health support, making art relevant for vulnerable youth populations (16).

### Arts-based interventions

Arts-based interventions have been used with a range of populations – including children dealing with traumatic events, special needs and disabilities, medical conditions, no clinical diagnosis, and those at higher risk, showing positive outcomes in all contexts (17). Research has demonstrated the effectiveness of arts-based interventions in enhancing self-awareness and facilitating emotional expression, as well as in promoting emotional regulation and reducing behavioral problems (18–20). Qualitative findings from a pilot study involving children with emotional and behavioral difficulties revealed that engaging in art created a safe space for self-expression, enabling children to feel empowered, reduce their perceived stress, and develop healthier coping mechanisms (21).

Previous literature review suggests that art activities have the potential to foster the development of identity, self-esteem and confidence, while also creating a sense of belonging. Collectively, these factors are argued to be protective for underprivileged and higher-risk youth (22). Art activities delivered within after-school programs have been associated with positive outcomes (15). For instance, a study conducted with low-income youth found that participation in art activities not only supported psychological well-being but also positively influenced academic outcomes by enhancing social skills, academic confidence and competence (16).

For children specifically, art offers a non-verbal medium through which they can express thoughts and emotions that may be difficult to articulate verbally (20). Art can also serve as a conduit through which children tell their stories, reframe their experiences, and create a sense of meaning in their lives (21). This can be particularly beneficial for youth facing adversity by giving them a sense of agency. For example, one study conducted with at-risk youth found that artmaking promoted hope and resilience, both serving as protective factors in the face of adversity (23).

### Arts-based interventions and psychological needs’ satisfaction

Arts-based interventions are commonly used approaches that can support basic psychological needs, including the need for autonomy, competence, and relatedness (24–26). Basic psychological needs’ satisfaction is rooted in self-determination theory (SDT), an empirically supported framework centered on human motivation, development, and well-being (27). It posits that individuals are inherently motivated to grow, learn, and develop within a supportive environment. According to SDT, satisfying the needs for autonomy, competence, and relatedness is essential for optimal functioning and well-being (27, 28).

The need for autonomy is defined as the need to control one's own choices and actions and to act in accordance with one's own values and interests (29). Engagement in art allows children to make choices about what to create and how to create it, thereby fostering a sense of agency and supporting their autonomy (26, 30). The need for competence is defined as the need to feel effective in one's environment and the ability to perform an act with some level of confidence (29). Art facilitates emotional expression by enabling children to communicate through symbolic forms, making it easier to convey complex thoughts and emotions (18). Additionally, participation in art activities helps children develop new skills and strengthen existing ones, contributing to a greater sense of competence (18). The need for relatedness is defined as the need to feel connected and accepted, and to experience a sense of belonging (29). Through artmaking, children can develop social skills, such as collaboration and empathy, while also connecting with peers who may share similar experiences, therefore nurturing their feeling of relatedness (31). Although findings from several studies highlight the potential of arts-based interventions to support the satisfaction of basic psychological needs, few studies have specifically examined this**.**

## Study aims

In collaboration with a community-based after-school program, the research team developed an arts-based intervention designed to address the needs of children participating in the program. The research question guiding this study was: *How can an arts-based intervention support the mental health and basic psychological needs of disadvantaged children?* The aims of the study were thus to explore the perceived benefits of this intervention for children's well-being and to document how participation in the intervention may support their basic psychological needs.

## Methodology

### Participants

Participants were recruited through an ongoing collaborative partnership between the research team and an after-school tutoring program offered by a community organization serving rural communities in a bilingual region of Quebec, Canada. The organization offers a range of programs aimed at fostering socialization, reducing isolation, and promoting academic achievement. Its after-school tutoring program serves elementary school-aged children in the region, with the primary goals of supporting academic success and reducing school dropout rates.

A convenience sampling method was used, as all children attending the after-school program were invited to participate in the intervention. The sample consisted of two groups of children (*N* *=* *32)*, aged 10 to 12 years, attending the after-school tutoring program and the two program coordinators. All children participated in the intervention while the program coordinators observed the workshops. Most children came from socioeconomically disadvantaged and low-education family backgrounds, and the sample presented psychosocial and developmental challenges, including emotional and behavioral difficulties, attachment insecurities, and learning disabilities. Limited mental health literacy and mental health-related stigmas were also observed within the sample. Parental consent was obtained before the intervention, and all children gave verbal assent to participate in the project. The study was approved by Bishop's University Ethics Board on December 20th, 2023 (#102681).

### Intervention

An 8-week arts-based intervention was implemented. The intervention was developed by a child clinical psychologist (CMH) and two psychology students (JP and KS), in collaboration with a researcher with training in dance and movement therapy (AM). Activities and themes were informed by previous work led by the research team, with activities that had been well received and appreciated by participants incorporated into the intervention (32). The intervention was also designed to align with the values and mission of the after-school program, which informed the selection of themes. The content of the intervention was reviewed and approved by the program coordinators before implementation. An overview of the intervention is provided in Table 1.

**Table 1:** Overview of art-based activities.

Week	Themes	Art medium
1	Beauty	Ugly drawing
2	Emotions	Dancing emotions
3	Love	Collage
4	Identity	Self-portait using Play-Doh or Legos
5	Friendship	Drawing of a friend
6	Fear	Play-Doh or Legos
7	Security	Building a “safe space” using Play-Doh or Legos
8	The future	Collective mural painting

Each weekly session lasted 30 minutes and followed a consistent structure: 20 minutes of artmaking followed by a 10-minute group discussion. During the artmaking periods, participants created individual pieces while being encouraged to interact with their peers, except for the mural-painting activity, which involved collaborative artmaking. During the discussion, participants were invited to share their artistic creations and reflect on the topic explored that week. The artistic activities served as prompts for the subsequent discussions and were selected to encourage introspection and self-expression.

The intervention was facilitated by the first author (JP), while observational data were collected by two research assistants (EM and MB). JP introduced the theme of each workshop and provided the instructions for the artmaking. Research assistants alternated between supporting the facilitation and recording observational notes, with one assistant actively assisting with the workshop while the other focused on note-taking. All three members of the research team interacted with participants throughout the sessions and were available to provide support during the artmaking activities and answer questions as needed.

### Data collection

In line with Braun and Clark's (33) qualitative approach, data were collected through four sources: semi-structured interviews (*n* = 14, average duration of 9 min) conducted with children after the intervention, one interview with the after-school program coordinators (duration of 57 min), as well as focus group discussions and observational data collected during the workshops. Children's artwork was not included in the analyses. This design was implemented to explore and report two facets of the intervention: children's subjective experiences and the coordinators’ external perspectives. All interviews and focus group discussions were audio-recorded for transcription and analysis. The interview guide was developed by the research team based on previous projects led by our team [ (24); see Appendix A]. The main themes of interest during the interviews with the participants were: i) the perceived benefits of the intervention on children's well-being, and ii) how the intervention supported children's sense of autonomy, competence, and relatedness. The interview with the coordinators documented their perceived benefits for children's mental health, along with any obstacles they identified during the workshops (see Appendix B).

The group discussions were led by the first author of the paper (JP) and lasted approximately 10 minutes. Each week, the discussion centered around a single theme and allowed participants to share their artistic creations, as well as their thoughts and feelings, and discuss the weekly topic as a group. The first author of the paper (JP) facilitated the discussion by redirecting questions to the participants to further guide and enrich the discussion. Two research assistants, undergraduate students in psychology (EM and MB), took extensive notes using an observational grid during weekly workshops (see Appendix C).

### Data analysis

An inductive-deductive thematic analysis was used in this study to identify themes reflecting participants’ perspectives on the intervention. Data analysis followed the six steps outlined by Braun and Clarke (33), while maintaining the flexibility necessary for themes to evolve throughout the coding process. All transcripts were analyzed using the MAXQDA2024 software. The first author (JP) generated the initial codes using an inductive approach. Subsequently, a deductive analysis informed by self-determination theory was undertaken to further organize and interpret the data according to the three basic psychological needs for autonomy, competence, and relatedness. A second coder (LD) independently analyzed the transcripts using the same coding tree. The coding tree was then reviewed, refined and used to develop and name the final themes. Data from focus groups and observational notes were integrated into the analysis and used to enrich the emerging themes from the interviews.

## Results

The thematic analysis yielded two overarching themes: perceived benefits of the intervention and satisfaction of basic psychological needs. These themes were divided into sub-themes, reflecting the perspectives of both the coordinators and the children who took part in the intervention.

## Perceived benefits

### Perceived benefits from the after-school coordinators’ perspective

Two sub-themes related to the perceived benefits of the intervention on children’s well-being emerged: 1) developing positive and secure relationships through trust and openness, and 2) developing socioemotional competencies and coping skills (see Table 2).

**Table 2:** Quotes from the after-school coordinators on perceived benefits.

Perceived benefits	Quotes
Developing positive and secure relationships	“They were able to talk and to stand up in front of everyone, and I was very impressed […] That's very good, and also good when you look at the degree of engagement and motivation […]”. (C1) “I was surprised to see them talk about sensitive stuff… I wasn't surprised by what they said you know, there are a bunch of things that I could have predicted, so I told myself it made sense, but I was surprised to see them share that with the group”. (C2) “I heard some kids share personal stuff that I didn't even know of, or other kids shared things that I would’ve never thought they would have in front of other kids.” (C1) “I know it, I see it, but now I hear it”. (C2) “They [children] were more open to talking with us about personal stuff following the intervention”. (C2) “It's certain that you grow, others will get to know you better, you will practice talking about yourself […], But mostly, the degree of attachment between the students, it changed the vibe between them”. (C1)
Development of socioemotional competencies and coping skills	“Even if it's not something that we can see from the outside, I think it [the activities], makes them feel good on the inside”. (C1) “To think about those kinds of things – even if it feels uncomfortable at first, is good for them. It gives them tools they can use later. When they experience an unpleasant emotion or find themselves in a situation where they don't feel well, maybe they’ll think back on the activities we did and what they learned. Maybe it will help soothe them or even help them resolve the problem.” (C1)

#### Developing positive and secure relationships through trust and openness

Both coordinators reported believing that the intervention created a safe and trusting environment that encouraged the development of positive and secure relationships, which were observed at three different levels: 1) the engagement with the research team, 2) the relationship with the coordinators, and 3) peer relationships.

One of the most significant changes observed over the course of the intervention was children's growing openness and trust towards the research team. At the beginning of the intervention, the coordinators had cautioned that many of the children typically found it difficult to engage with unfamiliar adults and that building trust often required time. This initial hesitation was noticed during the first workshop, when a child humorously shouted, “Stranger danger!”, upon seeing the research team. As the weeks progressed, however, observational data confirmed that the children became increasingly at ease in the team's presence. During the interviews, both coordinators mentioned that they were surprised to see the children openly share their personal stories, and they were impressed by how well several of them communicated. By the end of the intervention, the coordinators noted a marked shift in the children's comfort level, highlighting that many of them expressed sadness at the team's departure, indicating that a positive relationship had been established.

“With you guys, I think it's the most significant change […] to see at the beginning of the intervention compared with after eight weeks […] when they are sad to see you leave […] and to hear them say how much they had fun, and to see that they had become a lot more comfortable with you guys”. (C1)

Moreover, the coordinators reported being deeply moved by the vulnerability that children displayed and expressed gratitude for the safe and open space that allowed such sensitive topics to be explored. Being able to observe the workshops from a distance allowed the coordinators to get to know children on a more personal level, allowing them to gain new insights into their students. More specifically, the intervention opened a dialogue and offered a window into the children's inner worlds, which are often not accessible in the more structured context of the after-school program. Moreover, the children were more inclined to open up and felt more at ease discussing personal topics with them, outside of the intervention.

The supportive environment fostered by the research team throughout the workshops played a key role in encouraging children's engagement and fostering positive interactions between participants. Coordinators reported that children's interactions had positively evolved over the course of the intervention. For example, they noticed that during the group discussions following the artmaking, everyone's contributions were met with kindness. As mentioned by one of them: “Nothing negative or mean was said about someone else. We stuck to the positive and respectful” (C2), which contrasted with their typical social interactions. The activities allowed children to get to know each other better, and their participation in the workshops allowed them to connect:

“Even though we don't necessarily see the change [inside them], it's still happening. They [children] are more open with each other. The things that they learn about themselves […] and sometimes, when someone else says something, you can relate it to yourself – it helps you better understand how you feel, and how they feel too”. (C2)

#### Development of socioemotional competencies and coping skills

Both coordinators appreciated that the intervention supported the after-school program's mission to promote a positive environment for overall youth development. Although they reported not observing any immediate or noticeable behavioral changes in their students during the intervention, they believed that the workshops supported the development of the children's socioemotional competencies and coping skills. In their views, the activities provided valuable tools that the children could carry with them and apply in their daily lives.

The coordinators also noted how the intervention appeared to be accessible to all children. On one hand, the activities were adapted to their educational level and offered enough flexibility to be modified based on specific needs. On the other hand, the art-based approach was non-confrontational, allowing children to engage in self-expression and personal reflection at their own pace. Rather than feeling pressured, children could explore their thoughts and emotions safely and indirectly through the creative process and group discussions. One of the coordinators mentioned that:

“It [the intervention] is less confronting than talking with a therapist. They [children] don't have to sit down in an office and talk about their emotions. The same kind of work is done here, but indirectly, without the kids really noticing. This type of intervention is a lot more accessible to these kids; they would be resistant in a more rigid and structured environment.” (C1)

### Perceived benefits from the children’s perspective

Three sub-themes on the perceived benefits of the intervention were constructed in our analysis, which were primarily derived from the interviews with the children. They included: self-awareness and consciousness of others, emotional expression, and positive affect (See Table 3).

**Table 3:** Children's quotes on their experiences with emotional expression.

Perceived benefits	Quotes
Emotional expression	“It's really difficult for me to speak of my emotions, so if I can draw it or paint it, then I can share it with others” (participant 20). “It's like sometimes, you want to talk about it, but at the same time you don't really want to talk about it, so it's easier to just draw it” (participant 11) “I really liked the activities because instead of having to talk about how we feel, we can just draw it” (participant 14) “I really liked being creative while building Legos or drawing. We are doing things about our emotions, how we feel and stuff life that”. (participant 2) “I felt more free to tell the truth, well not the truth […] but I feel more free to talk about my emotions”. (participant 23) “I talk more often about my emotions with my friends than I did before”. (participant 23)

#### Self-awareness and consciousness of others

Based on the interviews with the participants, the intervention seemed to have provided meaningful opportunities for them to explore their thoughts and feelings. This subsequently appeared to make way for self-discovery and consciousness of others. For one participant, self-awareness was described as being authentic and true to their feelings: “We should always listen to our emotions and feel a little bit of everything. If you don't feel a certain emotion […], you have to feel it to be yourself” (participant 16). Using the self-portrait activity as an example, one participant highlighted artmaking as helpful for facilitating emotional awareness: “When we were creating our self-portrait, if we were feeling happy, we could make a smiley face, but if we were sad or mad, we could do a sad face” (participant 29). For others, self-awareness and consciousness of others emerged together. As one participant expressed: “The activities made me realize that it was easier to solve conflicts… and I also realized things about myself and about others” (participant 11). In their interview, one of the participants reflected on their life in general by reporting that during the workshop on security, they described realizing that it is important to appreciate what we have (i.e, being grateful). Moreover, the group discussions appeared to provide a space where children could learn about their peers and therefore help in the development of their consciousness of others. In the interviews, a few children commented on their appreciation of the social component of the intervention. For example, one participant said: “It was fun to see what others created”.

#### Emotional expression

Children reported appreciating the opportunity to artistically express themselves without having to verbalize how they felt or thought. The themes addressed during the workshops elicited a range of emotions (i.e., joy, gratefulness, peacefulness, sadness, fear, etc.), which participants were able to identify and process through both the artmaking and the group discussions. As one of the participants explained: “During the workshop on the safe space, I felt happy and free. But during the workshop on fear, I felt sad, and at the same time, I was scared” (participant 16).

Several participants noted that they found it difficult to verbalize their emotions. Using artistic mediums––such as drawing––appeared to make it easier for them to externalize and communicate their feelings. For others, the intervention did not seem to influence their ability to express their emotions. Indeed, when asked whether the intervention had benefited them, a few children responded “no” without further elaboration. Among those for whom the intervention facilitated emotional expression, the creative process not only allowed them to better understand their emotional state but also helped them feel understood by others.

A particularly meaningful moment occurred during the group discussion on the topic of love. The conversation naturally shifted toward self-love, prompting two children to share past experiences of struggling with negative self-perceptions. They disclosed moments when they had spoken to themselves in harmful ways or even engaged in self-injurious behaviors.

“When I was eight years old, I didn't like myself, and I was telling myself hurtful things… Then I would think to myself, why am I doing this […], then I stopped. Now, I don't do it anymore”.

“When I was younger, sometimes I didn’t like myself, and I would hit myself at times I didn’t like myself”.

Moreover, the intervention created a space where it was safe for children to share and talk about their emotions, which facilitated emotional expression beyond the scope of the workshops for some participants.

#### Positive affect

In their interviews, several children reported experiencing positive emotions during artmaking, including feelings of calm, peace, happiness and pride. One participant shared: “It made me feel good to talk about a place [referring to the workshop on safe space] that made me feel good and secure” (participant 23). For some participants, taking part in the workshops represented a fun time to clear their minds. For example, one child said: “At the beginning of the workshop, I was not concentrated because I had a bad day or whatever… But during and after the activities, I was happy” (participant 29). While some participants described occasionally arriving at sessions feeling tired or irritable after their day at school, they also expressed feeling as though they had left the workshops in a better mood. As one participant mentioned: “It [the activities] made me forget about my bad day at school, and I could just draw something and have fun with my friends” (participant 11).

### Psychological needs satisfaction

To better understand the children's experiences with the workshops, we asked them during the post-intervention interviews whether the intervention had an impact on their self-determination. This included asking questions about their feelings of autonomy, competence, and relatedness. In response, the feeling of autonomy was described as a form of freedom in the decision-making process, whereas competence was mostly associated with feeling proud of one's creation. Relatedness stood out as an element that appeared to drive artmaking through the process of collaboration. Notably, participants spoke of how the intervention supported their need for relatedness before the interviewer asked questions on this topic (see Table 4).

**Table 4:** Children's quotes on the intervention's impact on their basic psychological needs.

Perceived benefits	Quotes
Autonomy	“I love being able to use my creativity to do what I want” (participant 5) “Sometimes, at home or at school, I have to do things that I don't want to […], but here, when we do the activities, it's like we’re free” (participant 12) “The mural was my favorite activity because we were free to do what we wanted” (participant 5) “I liked it beause there weren't any rules, like it was more flexible, you guys simply asked us to participate in the activities seriously” (participant 14)
Competence	“I was proud because I am not usually good at art, but I did well in the workshops” (participant 5) “My grades in art got better this year” (participant 5) “I was hesitant to share my art with the group because I didn't think my drawing was good enough, I can be a little hard on myself…”(participant 8) “I was disappointed [with one of the activities] because my fine motor skills are not very good. But in the other activities, I felt better” (participant 31) “I really liked the activities because we were doing artistic stuff like drawing, and I am really good in arts, it's like my talent” (participant 11)
Relatedness	“I was happy to spend time with my friends, we were building and creating things, and playing, and laughing together” (participant 14) “My relationship with my classmates changed since the beginning of the intervention, because before I didn't really talk to anybody, and now suddenly, I feel respected by everyone, and I feel like I have the right to speak” (participant 5) “I used to only have one friend […] during the activities I made some more, and now I have several friends” (participant 11) “It improved my relationships with my friends.” (participant 11)

#### Autonomy

The concept of autonomy was related to one's perceived ability to make their own choices. At the beginning of the intervention, some participants turned to their peers or to the coordinators for inspiration on what to create. As the intervention progressed, however, children increasingly embraced their freedom to choose both the artistic medium and the content of their creation, thereby requiring less support. For others, having the freedom to create was welcomed and seen as an opportunity for creativity rather than as a difficulty. In their interviews, a few children mentioned that they particularly enjoyed the art activities because there were no strict rules to follow and that they could use their creativity. Aside from following the general theme, children appreciated being free to create what they wanted and how they wanted, which contrasted with a more traditionally structured environment they were accustomed to at school or even at home. Overall, participants noted that the intervention supported their sense of autonomy. However, many were not able to provide specific examples of how the intervention had supported their sense of autonomy.

#### Competence

The concept of competence was tightly linked with a sense of accomplishment and a sense of pride. For instance, one participant explained that they had never considered themselves good at art, but through practicing during the workshops, they felt their artistic skills had improved significantly. Another participant mentioned that their art grades had improved since taking part in the intervention. In contrast, a few participants acknowledged facing challenges with specific art mediums – whether due to a lack of ideas or feeling less skilled in areas like painting or dancing. In general, children reported liking activities they were competent at and disliking activities in which they felt incompetent.

#### Relatedness

The concept of relatedness emerged as a central theme in nearly all participant interviews and was frequently described as a helpful facilitator to the artmaking process. For example, several participants acknowledged open communication and collaboration with others as being most helpful for facilitating a satisfying artistic creation. When also asked what they appreciated most about the workshops, several students reported that they enjoyed taking part in an activity that was different from their usual routine, especially because they could do it alongside their friends. Observational notes further corroborated these findings; indeed, collaboration appeared to act as a critical component of this process during the artmaking and group discussions, where children often built upon each other's ideas. The intervention also seemed to have a positive influence on peer relationships beyond the scope of the workshops. A few participants reported that their friendships had improved over the course of the intervention, and one said they felt more respected by others.

## Discussion

The present study explored the perceived benefits of an 8-week arts-based intervention aimed at supporting the well-being of underprivileged children living in a rural area. Based on interviews with program coordinators and participants, thematic analysis suggested that the intervention appeared to create a safe and supportive space for children to explore their thoughts and emotions, connect with their peers and staff, and support their basic psychological needs. In contexts where access to traditional mental health resources is limited, these findings highlight art as a potential therapeutic modality for supporting underprivileged children's mental health.

## Perceived benefits

### Developing positive and secure relationships

The intervention appeared to foster positive and secure relationships between participants, their coordinators, and the research team. One key finding from the study was the progression from children's initial hesitation to gradually giving way to a sense of trust towards the research team. This trust developed through the provision of an accessible and psychologically safe space for children. This finding was deemed especially meaningful considering children's attachment insecurities and documented difficulties, which the program coordinators corroborated. Indeed, children's initial apprehension toward the research team may have reflected underlying attachment-related difficulties or simply stigma associated with discussing mental health and personal vulnerabilities. This finding suggests that the climate created throughout the intervention played a critical role in facilitating engagement. Previous research has documented similar results; participants who are initially apprehensive toward art-therapists tend to gradually become more forthcoming with them as the therapeutic alliance continues to strengthen (34).

It was important for us to establish a strong alliance with participants by responding to the children's mental health needs in real time. Bosgraaf (35) referred to *responsiveness* as a concept utilized in psychotherapy to enhance the therapeutic alliance and defined it as “interacting in a way such that the other is understood, valued, and supported in fulfilling important personal needs and goals”. In our study, this was done by adapting the activities based on children's emotional states upon arrival, remaining flexible in the delivery of the workshops, showing genuine interest in children's artistic creation, and providing support when needed. Some authors have suggested that this aspect of artmaking (i.e., the presence and availability of a facilitator) can mimic secure attachments between workshop facilitators and participants, which, in turn, facilitates children's engagement with the arts (36). By adapting the intervention to children's needs and fostering a safe environment, participants gradually built trust toward the research team, which seemed to enhance their engagement in the activities (i.e., active participation and engagement with the arts, being vulnerable in front of others, etc.).

As suggested by the results, the workshops seemed to have fostered opportunities for coordinators to get to know their students better, which also seemed to have contributed to improving their relationships with them. Artmaking appeared to play a facilitating role in gradually engaging with the themes of the workshops and initiating a reflection among children, thereby preparing the participants for the discussion that followed. This finding is supported by the literature; art is recognized as a non-judgmental and non-directive approach, which facilitates emotional expression for children who are less familiar with or uncomfortable verbalizing thoughts and feelings (18). Through engagement with the art, reflection on weekly themes and exchange in groups, the artmaking process acted as a bridge between vulnerability and emotional expression, therefore allowing participants to build positive relationships amongst themselves and with the program coordinators.

### Development of socio-emotional competencies and coping skills

Results revealed that coordinators did not notice any immediate or visible changes in their students. This contrasts with previous arts-based studies where teachers reported reductions in hyperactivity, behavioral problems and emotional difficulties (19, 20). However, the present findings nonetheless suggest the potential of arts-based intervention in supporting children's mental health, as the workshops appeared to provide participants with transferable skills that students could carry with them as they navigate future personal and interpersonal challenges. For underprivileged children with attachment or emotional and behavioral difficulties, learning effective emotional regulation and coping skills may be highly valuable as it fosters coping and resilience, while protecting them against mental health problems in the long term (37).

It is also important to highlight that participants in this study did not have access to other mental health resources in their community or at school. As a result of this lack of resources, the after-school program is often the main point of contact and trust for these children's psychological needs and well-being. Accordingly, the intervention may have served as a crucial entry point for mental health promotion and prevention.

### Emotional expression and positive emotions

The 8-week intervention appeared to enable some participants to develop improved self-awareness and consciousness of others, while being able to reflect on their emotions during the workshops. For instance, participants reported enjoying the use of diverse artistic mediums to externalize and express emotions, describing that this approach felt easier and more natural than verbal expression. This is consistent with existing literature on arts-based interventions, which emphasizes the value of creative processes in facilitating emotional expression and regulation (21, 38). Building on this finding, Moula (2021) suggests that artistic metaphors and imagery can support children in reflecting on their emotions and communicating them more effectively than through verbalization alone. Indeed, art-making may create a psychological distance from emotional content, making expression less threatening and overwhelming for children (18, 39).

Furthermore, participants reported experiencing positive emotions during the workshops, which may have directly contributed to their overall sense of well-being. Other studies also reported the emergence of positive emotions during artmaking (19, 40). The intervention may have thus provided a source of enjoyment and positive emotions, which is particularly important for underprivileged children, whose life circumstances may offer fewer opportunities for positive emotional experiences (31). In addition, by identifying and exploring their emotions, children may have been able to develop their emotional awareness, as previously mentioned. Previous research using arts-based interventions showed that by becoming more conscious of the complexity of their emotions, children can also become aware of conflicting emotions and learn that positive and negative emotions can coexist (e.g., happy, free, sad, fear) (21).

### Self-awareness and consciousness of others

Self-awareness and consciousness of others are two core competencies that develop throughout childhood and contribute to positive functioning and social skills that can be understood through the lens of social-emotional learning (SEL; 13). Self-awareness refers to “the ability to recognize one's emotions and thoughts and how they influence behaviors”, whereas social awareness refers to “the ability to take the perspective of and empathize with others” (41). The development of these competencies has been associated with a range of positive outcomes, including enhanced well-being, stronger interpersonal relationships and improved academic achievement (12). Moreover, they can also buffer the impacts of risk factors, highlighting their importance for underprivileged children (37).

In this study, self-awareness and consciousness of others appeared to be nurtured through artmaking and sharing, as children received opportunities to learn about themselves and others. Existing studies also support the use of art to foster introspection and self-discovery (21, 31). For instance, one study revealed that children were able to express themselves authentically, learn about their ‘true self’, and feel more comfortable showing their ‘true self’ to others after participating in an arts-based intervention (42). Indeed, exploring one's emotions and creatively reflecting on oneself can help children develop self-awareness; evidence even suggests that it could also improve self-esteem and self-acceptance (31, 43).

Group discussions, as reported by the children and coordinators of this study, appeared to foster a space where participants could learn about their peers, develop mutual understandings and consciousness between each other, while also building a sense of connection. According to SEL, social awareness is a pillar for building positive and meaningful relationships (13). The welcoming of differing viewpoints within the group may have contributed to this process, encouraging both self-reflection and the appreciation of diverse perspectives (40). Future studies should investigate how and to what extent arts-based interventions can foster the development of social and emotional competencies.

### Psychological needs satisfaction

The workshops, which were intentionally designed to create collaborative artmaking, group discussions, and shared reflections, appeared to promote a sense of relatedness, suggesting the potential for art to foster children's basic psychological needs. Other studies have reported similar findings; art is a way of connecting with others and relating to those who live similar situations to ours (31). Sharingone's artwork can also help normalize children's experiences and feelings, making them feel like they are not alone, which is especially important for those who might not get this validation at home or at school (31). Importantly, findings from this study could suggest that the intervention had a positive influence on peer relationships that extended beyond the workshops themselves, including improved interpersonal relationships and stronger friendships. Considering that the after-school program's mission is in part based on fostering positive relationships, this result was especially valuable for the program coordinators. Moreover, peer relationships and support are strong protective factors against mental health problems, especially for children who receive low school and/or family support (44). In the present study, children's experience of relatedness was closely tied to positive emotions shared through collaboration during artmaking, which further shows the potential of arts-based interventions for strengthening relatedness among underprivileged children.

The intervention also appeared to support children's need for competence, which was closely tied to feelings of accomplishment and pride in children's artistic creations. Although the goal of arts-based interventions is process-oriented rather than product-oriented - that is, the focus is centered around the process of artmaking rather than on the result of the final creation - many children expressed howproud they were of what they had created (i.e., of the final product). This could be explained by the fact that when children engage in artmaking – typically at school – they are evaluated and subject to their teacher's expectations (25). Feelings of accomplishment were particularly meaningful for those who initially perceived themselves as “not good” at art but later saw that they improved after repeated participation. This suggests that children may have experienced a sense of mastery, which in turn reinforced their motivation and confidence to engage in artistic activities (27). Moreover, artmaking could also have supported children's need for competence by facilitating emotional expression, making it easier for them to externalize their thoughts and feelings (24). By supporting children's sense of competence, artmaking could also directly support their well-being, as competence is a strong predictor of well-being in school-aged children (28).

Compared to findings of self-perceived benefits in competence and relatedness, expressions of autonomy were more subtle. Yet, findings showed that the intervention nonetheless supported children's need for autonomy in some ways. In the present study, children reported that the availability of a variety of artistic mediums supported, to some extent, their need for autonomy. This is consistent with previous studies, where children perceived the choice of the art material as prompting decision-making and having a sense of control (24, 32). Decision-making is a reinforcer of children's autonomy, which might explain why they particularly enjoyed having the freedom to choose the material and the content of their creation (40, 45). Our findings showed that having the freedom to choose the art material was also linked to increased feelings of competence. This finding highlights the importance of incorporating a variety of artistic mediums within such interventions to support children's sense of autonomy, but also to accommodate different preferences and abilities, therefore ensuring that all participants have opportunities to experience a sense of accomplishment (24). Moreover, several participants appreciated the flexibility of the sessions, which contrasted with the more structured format experienced at school or at home, and helped in creating an autonomy-supportive environment. Previous research has shown that, in addition to environmental stressors, disadvantaged youth experience rigid expectations and have limited control over their situation (39). Considering this, the workshops may have served as rare opportunities in which children could exercise their autonomy.

## Strengths and limitations

This study has notable strengths. Most significantly, this research provided an opportunity for underprivileged youth to constructively explore and discuss emotions and mental health, a topic that is seldom provided to this demographic and often filled with stigmatization (16). Following these workshops, participants and coordinators of the after-school program highlighted how they perceived benefits in terms of the mental health of the children by receiving such an opportunity. Moreover, unlike other traditional arts-based intervention studies, this research provided participants with the additional opportunity of openly discussing their artistic creations in a group discussion format, thereby creating the additional unique contribution of this research: participants and the after-school coordinators attested to the potential value not only of artmaking, but also of discussing it.

Although this study has its strengths, it is not without its limitations. Most notably, the relatively short time allocated for each workshop (30 min; 8 total) largely limited us in our capacity to provide participants with the required time to constructively and fully explore each intervention theme in a manner that was satisfactory to their mental health needs. For example, some participants did not always finish their creations, and group discussions were sometimes cut short, thus potentially leaving some unsatisfied because they did not receive the opportunity to fully address their concerns related to the theme of the workshop. In our team's prior research, this type of intervention was traditionally implemented over longer sessions (60 min) to allow more time for developing artistic creations and extended group discussions (24, 46). However, in this study, we sought to inquire whether similar benefits could be experienced in a setting where time constraints are enforced. Our findings show potential for this intervention method to be effective; however, no control group was used. Further quantitative and longitudinal research is required before we can conclusively state whether these interventions can yield long-term benefits or whether such findings are applicable across varied populations or cross-cultural settings. On this note, and given the qualitative nature of this research, we are limited in our capacity to make generalizing statements regarding the broader impact of this intervention. Although qualitative research does not seek to generalize its findings across populations, we nonetheless caution readers against interpreting these findings as applicable to other clinical contexts, demographics, or cultural settings, such as underprivileged children in similar communities.

Lastly, due to the strong rapport that researchers had with both the participants and after-school program coordinators, the findings of this study may also have been at a higher risk of social desirability bias. Indeed, respondents of the semi-structured interviews may have felt inclined to speak positively about the intervention instead of disclosing their true and honest thoughts, which may have risked them over-reporting the benefits and under-reporting the negative aspects of the intervention. Although the risk for social-desirability bias is always present, we attempted to minimize these potential occurrences by reminding all interviewees (i.e., child participants and the afterschool program coordinators) that we were seeking only truthful responses–especially if they were negative–and that all forms of feedback were helpful.

## Implications and directions for future research

This study highlights the potential value of arts-based interventions as a medium for fostering children's well-being and satisfaction of basic psychological needs. Based on our thematic analysis, study results indicate that participants found art to serve as an accessible method for engaging and reflecting on emotions in a non-confrontational manner. These findings pose broader implications for underprivileged populations where mental health literacy is limited. Additionally, our findings suggested that having the ability to explore and engage freely in the art activities appeared to provide the children of this study with a sense of autonomy, competence and relatedness, thus highlighting the potential of such interventions to support their self-determination and well-being. Arts-based interventions appear to be one suitable setting for supporting children's mental health; however, further considerations should be made on how they can be best navigated under the specific conditions and parameters of after-school programs, such as time constraints. Additionally, from a prevention perspective, promoting the development of socio-emotional competencies through arts-based interventions could serve as a protective factor against future mental health difficulties.

With these implications in mind, future research should continue examining whether arts-based interventions can be effectively implemented within a 30-minute timeframe, as opposed to the traditional one-hour format. Furthermore, employing quantitative randomized controlled trial designs that assess pre- to post-intervention changes in mental health outcomes may provide greater insight into the mechanisms underlying observed improvements in well-being. The use of mixed-methods approaches, integrating both qualitative and quantitative data, would also strengthen understanding of participants’ experiences while allowing for the evaluation of measurable outcomes. Finally, cross-cultural investigations are needed to establish the generalizability of these interventions and provide more robust evidence regarding their capacity to promote well-being among underprivileged children across diverse settings.

## Data Availability

The datasets presented in this article are not readily available because confidentiality. Requests to access the datasets should be directed to jpiche20@ubishops.ca.
